# Comparison among Neuroblastoma Stages Suggests the Involvement of Mitochondria in Tumor Progression

**DOI:** 10.3390/biomedicines11020596

**Published:** 2023-02-17

**Authors:** Stefano Cagnin, Tomas Knedlik, Caterina Vianello, Ana Paula Magalhães Rebelo, Agnese De Mario, Marta Giacomello

**Affiliations:** 1 Department of Biology, University of Padua, Via U. Bassi 58/B, 35121 Padua, Italy; 2 CIR-Myo Myology Center, University of Padova, 35121 Padua, Italy; 3 Department of Biomedical Sciences, University of Padua, Via U. Bassi 58/B, 35121 Padua, Italy

**Keywords:** neuroblastoma, cancer, differentially expressed genes, cytoskeleton, microtubules, mitochondria, endoplasmic reticulum, mitochondrial dynamics, mitochondria–endoplasmic reticulum contact sites, MERCS, mitochondria-associated membranes, MAMs

## Abstract

Neuroblastoma (NB) is the most common extracranial tumor of early childhood and accounts for 15% of all pediatric cancer mortalities. However, the precise pathways and genes underlying its progression are unknown. Therefore, we performed a differential gene expression analysis of neuroblastoma stage 1 and stage 4 + 4S to discover biological processes associated with NB progression. From this preliminary analysis, we found that NB samples (stage 4 + 4S) are characterized by altered expression of some proteins involved in mitochondria function and mitochondria–ER contact sites (MERCS). Although further analyses remain necessary, this review may provide new hints to better understand NB molecular etiopathogenesis, by suggesting that MERCS alterations could be involved in the progression of NB.

## 1. Introduction

Neuroblastoma (NB) is a heterogeneous malignancy originating from the embryonic cells composing the neural crest [1,2]. NB is the most widespread extracranial solid tumor in children, accounting for nearly 10% of all childhood cancers [3,4]. NB is also the leading cause of cancer death in children younger than 5 years old, accounting for 15% of all pediatric cancer fatalities [5]. About 90% of NB cases are diagnosed before 5 years of age, while a third of those are diagnosed within the first year of life [4,5]. On the other hand, NB is a comparatively rare cancer with an incidence of 10.2 cases per one million children younger than 15 years; it affects 12 infants per 100,000 births [4,5,6].

NB belongs to embryonal neuroendocrine tumors of the peripheral nervous system [2]. It originates from the neural crest progenitor cells, particularly from the sympathoadrenal cell lineage [7]. Therefore, NB can grow anywhere along the sympathetic nervous system. The majority of NB (65%) develops in the abdomen, usually originating in the adrenal gland [8]. However, other sites of NB include the chest (20%), the neck (5%), or pelvis (5%) [8]. Due to a number of primary tumor locations, NB exhibits heterogenous biological, clinical, and morphological characteristics. Clinical symptoms of NB are often vague (e.g., fever, fatigues, loss of appetite) and diverse, depending on the site of malignancy, such as constipation and abdominal distention (NB in the abdomen) or breathing problems (NB in the chest) [9]. Similarly, the clinical outcomes of NB are variable, ranging from spontaneous regression to major treatment-resistant metastatic cancer with poor patient prognosis [10].

NB is classified (by the International Neuroblastoma Staging System) into six stages, in relation to the outcome of surgery used to remove the tumor [11]. In our work, we focused on three stages: (i) stage 1: the tumor is localized in the area of its origin and can be completely removed by surgery; (ii) stage 4: the tumor is widespread to distant lymph nodes, bone marrow, skin, liver, or other organs (except as defined for stage 4S); (iii) stage 4S (applicable only to infants under 1 year of age): the tumor mass locates as defined for stage 1, but with propagation limited to skin, liver, or bone marrow [11].

The pathogenesis of NB and biological processes leading to NB development from normal cells within the neural crest have not been fully described and elucidated. Only little is known about the potential processes and biological pathways that may lead to the initiation, development, and progression of NB. However, it seems there is no direct cause of NB pathogenesis; it rather requires multiplicity and cooperation of several effects, leading altogether to tumorigenesis [2]. The vast majority of neuroblastoma is sporadic and non-familial [12]. Only approximately 1% of cases are familial; germline mutations in several genes, such as anaplastic lymphoma receptor tyrosine kinase (*ALK)*, paired mesoderm homeobox protein 2B (*PHOX2B)*, and kinesin family member 1B (*KIF1B)* have been identified in patients with familial NB [12,13]. Interestingly, defects in *ALK* (anaplastic lymphoma kinase) genes have been demonstrated to occur in about 15% of NB cases [13,14]. Additionally, only the activating mutations in *ALK* and amplification of v-myc avian myelocytomatosis viral oncogene neuroblastoma-derived homolog (*MYCN)* have been shown to be oncogenic de novo in mice [15,16,17]. The presence of *MYCN* oncogene amplification highly correlates to advanced NB stages [17]. *MYCN*, a member of the myc proto-oncogene family, acts as a transcriptional factor for control of cellular differentiation and proliferation and plays an important role in the survival of neuroblastoma cells.

Mitochondria are dynamic organelles responsible for several cellular functions. They establish complex networks in the cells that can rapidly rearrange to react to the needs and metabolic state of the cell, through specific mitochondria fission and fusion processes [18,19,20,21]. Mitochondrial dynamics is regulated by their interactions with other organelles as well as the cellular cytoskeleton [18,22,23]. Evidence of the former are mitochondria–endoplasmic reticulum (ER) contact sites (MERCS), in which their surfaces are separated by a 10–80 nm gap [24,25,26,27,28,29]. Importantly, MERCS regulate crucial cell processes [27,30], such as Ca^2+^ and lipid homeostasis [31,32,33], mitochondrial fission [34], and apoptosis [35]. Due to their valuable role in mitochondrial biology and dynamics, MERCS have recently gained careful attention from biologists; however, all of their biological functions have not yet been fully described [36]. Similarly, mitochondria can also directly associate with microtubules and actin, the components of the cytoskeleton [22]. While microtubules serve for long-range mitochondria transport, the actin filaments regulate rather short-distance mitochondrial movement [37], their docking [38], and fission [39,40].

Mitochondria play a valuable role in the control of several crucial cellular processes, such as calcium homeostasis [41,42], ATP production [43,44,45], and apoptosis [46,47]. Thus, it is not surprising that mitochondrial dynamics dysfunction has been linked with the development and/or progression of tumors [48] including glioblastoma [49,50], melanoma [51], hepatocellular carcinoma [52], and pancreatic [53], breast [54], ovarian [55], and prostate cancers [56]. Regarding NB, interestingly, Çoku et al. has associated the reduction in the number of MERCS with aggressive NB displaying chemoresistance. This suggests that decreased mitochondria–ER interaction promotes neuroblastoma multidrug resistance [57]. Hence, the above-mentioned pieces of knowledge pose the challenging question of whether MERCS participate in NB development and progression.

To tackle this question, we used previously published datasets to identify genes differentially expressed in non-metastatic (stage 1) and metastatic neuroblastoma (stage 4 + 4S, which are characterized by metastases on skin, liver, and bone marrow) involved in MERCS structure and mitochondrial dynamics. We found that MERCS proteins previously shown to alter several aspects of cancer progression, i.e., cell metabolism, proliferation, and division, are upregulated in NB stage 4 + 4S, indicating the possibility that MERCS could be involved in the progression of this malignancy.

## 2. Materials and Methods

We used the GEO dataset GSE45547. This dataset includes 649 NB tumor samples whose gene expression was studied by a single-color Agilent-020382 Human Microarray composed of 44 K oligonucleotide probes. Tumor samples were classified according to the International Neuroblastoma Staging System. Data from NB stage 1 (153 samples) have been compared to stage 4 + 4S (292 samples). Raw data were logarithmic-scaled and quantile-normalized using the R Limma 3.26.8 package [58] in the R suite (Supplemental Figure S1); normalized data were used to calculate differentially expressed genes (DEGs) using the R Limma 3.26.8 package, which bases the identification of DEGs on linear models. The Benjamini–Hochberg false discovery rate method was used to correct for multiple tests and only genes with adjusted *p*-values under 0.05 were considered differentially expressed. Differentially expressed genes were classified according to Gene Ontology (GO) definitions using the WEB-based GEne SeT AnaLysis Toolkit [59], correcting statistical significance for multiple tests with the Benjamini–Hochberg method, considering enrichment significant when FDR < 0.05, and using 5 as the minimum number of IDs in the category and 2000 as the maximum number of IDs in the category to allow the consideration of categories. GO terms are either close in the GO hierarchy (sibling terms) or are related by inheritance (child and parent terms). Therefore, they may consist of a redundant list difficult to interpret. Revigo is a web tool used to reduce GO redundancy [60]. The tree map implemented in the Revigo web tool was used to summarize GO categories. We maintained default parameters (remove absolute GO terms, SimRel as semantic similarity measure) and used *Homo sapiens* as a species to be used. GO terms and corresponding *p*-values were considered for analyses with the Revigo web tool.

## 3. Results

**Figure 1:**
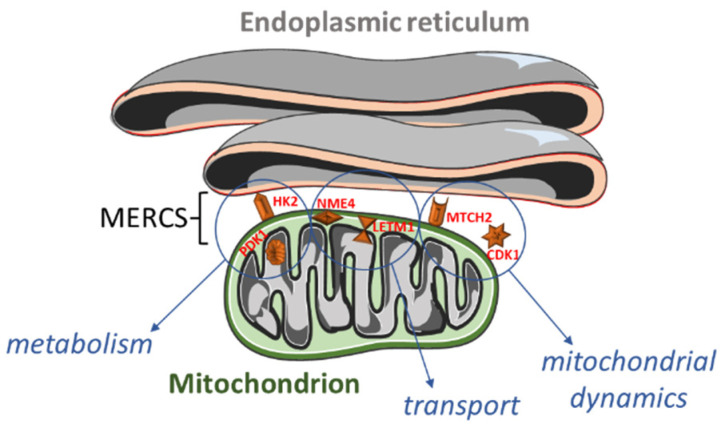
Overview of the proteins localized within or close to mitochondria–endoplasmic reticulum (ER) contact sites (MERCS) and their functions. Hexokinase 2 (HK2; outer mitochondrial membrane) and pyruvate dehydrogenase kinase 1 (PDK1; mitochondrial matrix) are involved in the (carbohydrate) metabolism. NME/NM23 nucleoside diphosphate kinase 4 (NME4; mitochondrial intermembrane space) and leucine zipper and EF-hand-containing transmembrane protein 1 (LETM1; inner mitochondrial membrane) participate in the cardiolipin and calcium transport. Finally, mitochondrial carrier 2 (MTCH2; outer mitochondrial membrane) and cyclin B1-dependent kinase 1 (CDK1; cytoplasm, mitochondrion, nucleus) regulate mitochondrial dynamics.

**Figure 2:**
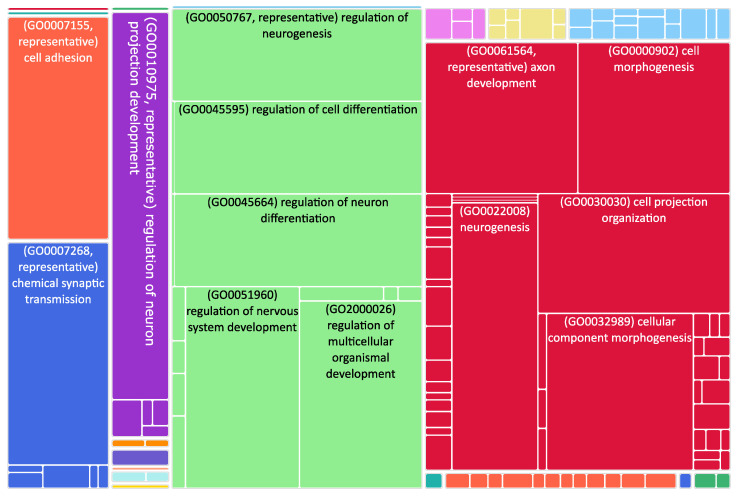
Tree map of downregulated genes in the stage 4 + 4S. Each rectangle is a single cluster representative. The representatives are joined into “superclusters” of loosely related terms, visualized with different colors. Sizes of the rectangles are adjusted to reflect the *p*-value of the GO term (“biological process”). Most representative terms are indicated. Several terms associated with neuronal function are enriched.

**Figure 3:**
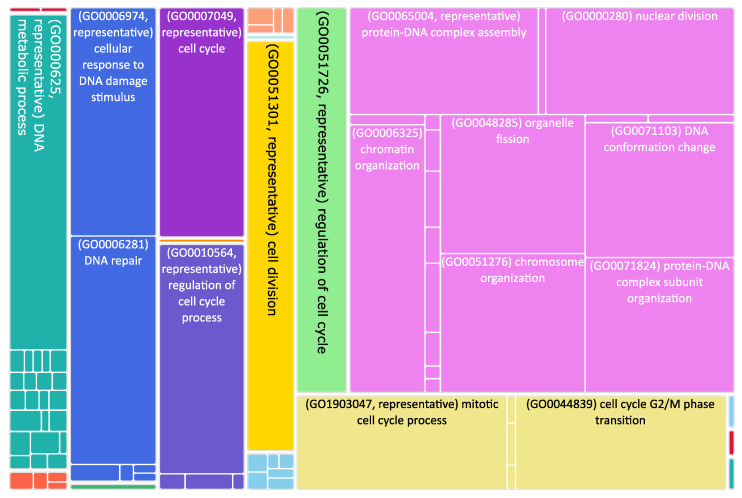
Tree map of upregulated genes in the stage 4 + 4S. Each rectangle is a single cluster representative. The representatives are joined into “superclusters” of loosely related terms, visualized with different colors. Sizes of the rectangles are adjusted to reflect the *p*-value of the GO term (“biological process”). Most representative terms are indicated. Upregulated genes in the stage 4 + 4S are prevalently associated with cell cycle regulation.

The role of mitochondrial dynamics in NB progression is still poorly explored, despite a reduction in the number of MERCS being associated with aggressive NB phenotypes characterized by chemoresistance. To study their involvement in NB progression, we took advantage of published transcriptomic datasets and analyzed the expression changes occurring during NB progression. Using NB stage 1 as control, we found out that 1971 genes were upregulated in stage 1 and 1529 were upregulated in stage 4 + 4S (Supplemental Table S1 and Figure 1). Interestingly, we found that genes involved in the movement of subcellular vesicles, in neurogenesis, and in synaptic signaling were downregulated in stage 4 + 4S compared to controls (Figure 2 and Supplemental Table S2). At variance, upregulated genes were involved in the control of the cell cycle (Figure 3 and Supplemental Table S2). This is in agreement with the evidence that suggests dysregulation of the cell cycle results in NB formation [61]. Of note, various mechanisms involved in the control of cell cycle progression dictate whether NB cells undergo neural differentiation or enter into cell cycle arrest and adopt senescence-like state [62]. 

**Figure 4:**
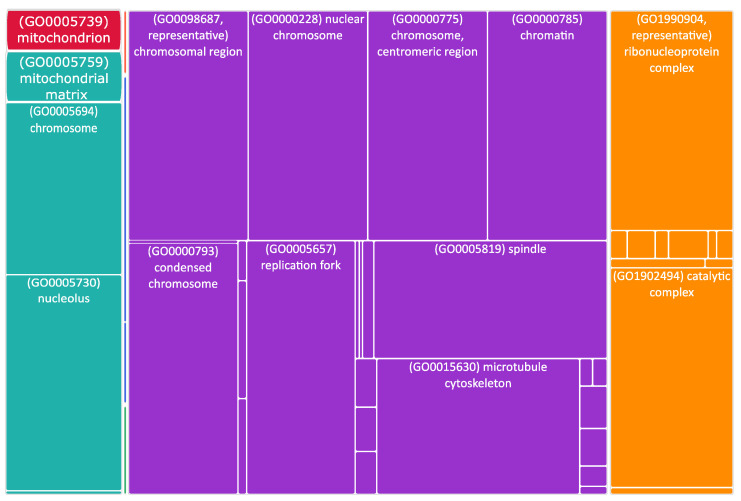
Tree map of upregulated genes in the stage 4 + 4S. Each rectangle is a single cluster representative. The representatives are joined into “superclusters” of loosely related terms, visualized with different colors. Sizes of the rectangles are adjusted to reflect the *p*-value of the GO term (“cellular component”). Most representative terms are indicated. Cytoskeleton and mitochondria are enriched terms.

A significant number of altered genes encoded for cytoskeletal proteins, or localized in mitochondria or in the endoplasmic reticulum (Figure 4 and Supplementary Table S3). As speculated, we also found some differentially expressed genes (DEGs) encoding for MERCS proteins (Figure 1).

Among the identified MERCS DEGs, HK2 (hexokinase 2) was 1.4 times upregulated in stage 4 + 4S (Supplemental Table S1). It is interesting to note that PDK1 (pyruvate dehydrogenase kinase 1) was also upregulated. PDK1 and HK2 are involved in cell metabolism, which is known to be affected in neuroblastoma. Indeed, in N-Myc (*MYCN*)-amplified NB cells, altered energy metabolism due to direct or indirect activation of genes involved in glycolysis, glutamine and fatty acid metabolism, and mitochondrial dysfunction has been reported [63]. It has been shown that PDK1 is specifically required for the metabolic response to hypoxia and nutrient deprivation in some cancer types; PDK1 phosphorylates pyruvate dehydrogenase (PDH) to inhibit its activity, thereby reducing the level of pyruvic acid in the tricarboxylic acid cycle, which affects the rates of oxidative phosphorylation [64]. The outcome of upregulated PDK1 activity on metabolic reprogramming might be amplified by the concomitant increase in HK2 expression observed in stage 4 + 4S (Supplemental Table S1). HK2 catalyzes the first step of glycolysis and it has been found upregulated in several types of cancer; its activity has been associated with the Warburg effect, e.g., high lactate production in the presence of oxygen [65]. Notably, upregulation of PDK1 and HK2 has been associated with an increased proliferation and poor prognosis in *MYCN*-amplified NB [66]. 

As mentioned before, many upregulated genes in grade 4 + 4S were involved in the regulation of the cell cycle (Figure 3). Interestingly, several stages of the cell cycle are accompanied and/or controlled by changes in mitochondria dynamics; for example, the cell cycle regulator CDK5 (cyclin-dependent kinase 5) is necessary for mitochondrial movement [67]. Interestingly, increased CDK5 activity was shown to be involved in several cancers [68,69,70]. Here, we found that CCNB1 (cyclin B1) and CDK1 (cyclin-dependent kinase 1) were twice upregulated and NME4 (NME/NM23 nucleoside diphosphate kinase 4) was 1.6 times upregulated (Supplementary Table S2). CDK1 is essential for cell division in mammals; CDK1 combines with cyclin B1 to form the cyclin B1–CDK1 complex, which is required for early mitotic events such as spindle assembly, nuclear envelope breakdown, and chromosome condensation. Genomic aberrations of cyclin B1 and CDK1 genes are associated with a dysregulated G1 entry checkpoint and have been described in NB [71]. NME4 belongs to a multifunctional NDPK/NME protein family. Its members are predominantly found in the mitochondrial intermembrane space, tethered to the inner membrane via anionic phospholipids, such as cardiolipin (CL) [72]. Here, NDPK-D plays two roles crucial for proper mitochondrial physiology. Firstly, it transfers phosphate from ATP (generated by oxidative phosphorylation pathway) to other NDPs (mostly to GDP). This phosphotransfer reaction, thus, generates GTP necessary for powering mitochondrial GTPases, such as optic atrophy 1 (OPA1) [73], a dynamin protein mediating mitochondrial fusion and maintaining the cristae. Secondly, NDPK-D transports CL from the inner mitochondrial membrane to the outer one; there, CL functions as a pro-apoptotic or pro-mitophagic signal.

NME4 inhibition was recently shown to reduce NB cell migration and to be involved in NB cell differentiation [74]. However, further research needs to be undertaken both to validate the functional NME4 role in NB pathogenesis and to identify the kinase targets and signaling pathways regulated by NME1. If elucidated and confirmed, NME4 and its activity may represent a novel target for NB therapy by inducing NB cell differentiation [75,76,77].

Other mitochondria genes differentially expressed in high-stage NB are LETM1 (leucine zipper and EF-hand-containing transmembrane protein 1; 1.4 times upregulated in stage 4 + 4S) and MTCH2 (mitochondrial carrier 2; 1.5 times upregulated in stage 4 + 4S; Supplemental Table S1). 

LETM1 is a mitochondrial proton/calcium antiporter that has been described to mediate proton-dependent calcium efflux from mitochondria [78]. Mitochondria calcium handling is fundamental not only for their activity, but for the overall cell physiology, fostering either ATP production in case of physiological calcium uptake, or promoting permeability transition in case of calcium overload [32,35]. 

LETM1 is also crucial for the maintenance of mitochondrial tubular networks and for the assembly of respiratory chain super-complexes [79]. LETM1 knockdown caused mitochondria to become dot-like structures, losing their tubular networks to an extent significantly greater than that observed in OPA1-knockdown cells. Images of mitochondria lacking LETM1 were reminiscent of observations following overexpression of pro-fission proteins such as Fis1 or knockdown of pro-fusion proteins such as OPA1 [80,81]. Although the functions and mechanisms of LETM1 with respect to cell viability and tumorigenesis remain controversial, accumulating data suggest that LETM1 is a crucial candidate. Deepening its role will clarify how mitochondria regulate the normal life of the cell and tumor-associated metabolic reprogramming. 

MTCH2 is an outer mitochondrial membrane protein that functions in the process of intrinsic cell death as well as in the regulation of fatty acid metabolism. MTCH2 interacts with the truncated BH3-interacting domain death agonist (tBID) to regulate cell apoptosis [82]. Previous studies demonstrate that loss of MTCH2 impairs mitochondrial architecture and functions, including enlarged size [83], reduced motility [84], and elevated oxidative stress [85]. MTCH2 expression was associated with several types of tumors. 

Altogether, our data suggest that several MERCS-resident proteins, regulating (carbohydrate) metabolism, mitochondrial dynamics, and molecular transport, may be involved in the etiopathogenesis of NB.

## 4. Discussion

Neuroblastoma is the most common extracranial tumor of early childhood and accounts for 15% of all pediatric cancer mortalities. This tumor is characterized by high clinical and biological heterogeneity. Indicating several genetic aspects, besides external factors, might cooperate to define the phenotypic outcomes. A tremendous effort has been made to elucidate the molecular mechanisms implicated both in the etiology and pathogenesis of NB, which eventually resulted in identification of novel therapeutic targets. Whole-genome-based methods, such as high-throughput genome analysis, genome-wide association studies, and genome sequencing, have revealed genetic alterations and disrupted pathways that participate in NB growth and development. However, the precise pathways and genes involved during NB progression are still largely unknown. Metabolic reprogramming accompanies development and progression of many cancer types; thus, mitochondria, which are central for cells’ energy production, fundamentally contribute to tumorigenesis. A great deal of evidence in the last years has shown that the shape of these organelles, along with their interplay with other subcellular compartments, control their function and energy production ability. 

In the context of NB, the role of mitochondria in shaping the phenotypic outcomes and progression of the disease has not been fully elucidated. Here, we performed a differential gene expression analysis of NB stage 1 and stage 4 + 4S to discover NB-related biological processes driven by mitochondrial dynamics. Among many differentially expressed genes, we found some interesting genes coding for proteins either residing at mitochondria or modulating their function: HK2, PDK1, NME4, LETM1, MTCH2, and CDK1. Based on the current literature, we propose and explain that these proteins could cooperate to define the metabolic adaptations needed to sustain cancer progression based on enhanced glycolysis and to promote cell cycle progression. 

Although further analyses remain necessary, this brief report provides new hints in NB molecular etiopathogenesis, suggesting that alterations of mitochondria dynamics could participate in the development and worsening of the disease.

## Data Availability

Gene expression data are available at GEO database (GSE45547).

## Supplementary Materials

The following supporting information can be downloaded at: https://www.mdpi.com/article/10.3390/biomedicines11020596/s1, Table S1: Differentially expressed genes; Table S2: Gene Ontology analysis based on Biological Processes; Table S3: Gene Ontology analysis based on Cellular Components. Figure S1: Normalized data used to identify DEGs. In green stage 1 tumor samples and in violet stage 4 + 4S. Total number of samples (box plots) is 445. This makes difficult the visualization of the sample names but it is possible to see that the median of the fluorescence intensity is the same for all the samples (lane in the middle of each box).
