# Supplementary material for: Comparison among Neuroblastoma Stages Suggests the Involvement of Mitochondria in Tumor Progression
Source: Biomedicines. 2023 Feb 17;11(2):596. doi: 10.3390/biomedicines11020596 (PMC9953471; doi:10.3390/biomedicines11020596)
Supplement: Supplementary file 1 [file biomedicines-11-00596-s001.zip › Supplemental Figure S1.pdf]

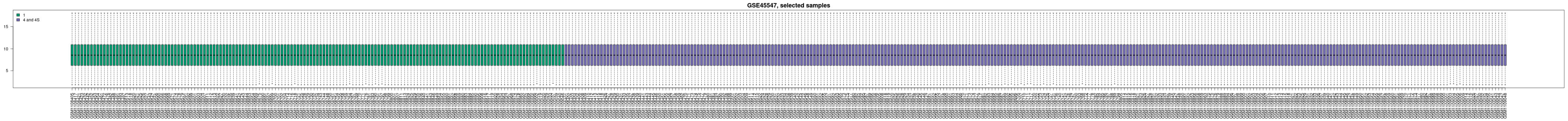


**Supplemental Figure S1.** Normalized data used to identify DEGs. In green stage 1 tumor samples and in violet stage 4 + 4S. Total number of samples (box plots) is 445. This makes difficult the visualization of the sample names but it is possible to see that the median of the fluorescence intensity is the same for all the samples (lane in the middle of each box).
